# Clinical features of migraine with onset prior to or during start of combined hormonal contraception: a prospective cohort study

**DOI:** 10.1007/s13760-021-01677-3

**Published:** 2021-04-29

**Authors:** Gabriele S. Merki-Feld, Peter S. Sandor, Rossella E. Nappi, Heiko Pohl, Christoph Schankin

**Affiliations:** 1 Department of Reproductive Endocrinology, University Hospital Zürich, Frauenklinikstrasse 10, CH-8091 Zürich, Switzerland; 2 RehaClinic Group, Bad Zurzach, Switzerland; 3 University of Zurich, Zürich, Switzerland; 4 Center for Reproductive Medicine, Gynecological Endocrinology and Menopause, Obstetrics and Gynecology Unit, IRCCS S. Matteo Foundation, Pavia, Italy; 5 Department of Clinical, Surgical, Diagnostic and Pediatric Sciences, University of Pavia, Pavia, Italy; 6 Department of Neurology, University Hospital Zurich, Zürich, Switzerland; 7 Department of Neurology, Inselspital, Bern University Hospital, University of Bern, Bern, Switzerland

**Keywords:** Migraine, Contraception, Hormone withdrawal, Menstrual migraine, Family history

## Abstract

Many studies have described the features of menstrually related migraines but there is a lack of knowledge regarding the features of migraine in combined hormonal contraceptive users (CHC). Hormone-withdrawal migraines in the pill-free period could differ from those in the natural cycle. Gynaecologic comorbidities, like dysmenorrhea and endometriosis, but also depression or a family history might modify the course of migraine. A better understanding of migraine features linked to special hormonal situations could improve treatment. For this prospective cohort study, we conducted telephone interviews with women using a CHC and reporting withdrawal migraine to collect information on migraine frequency, intensity, triggers, symptoms, pain medication, gynaecologic history and comorbidities (*n* = 48). A subset of women agreed to also document their migraines in prospective diaries. The mean number of migraine days per cycle was 4.2 (± 2.7). Around 50% of these migraines occurred during the hormone-free interval. Migraine frequency was significantly higher in women who suffered from migraine before CHC start (5.0 ± 3.1) (*n* = 22) in comparison to those with migraine onset after CHC start (3.5 ± 2.1) (*n* = 26). Menstrually related attacks were described as more painful (57.5%), especially in women with migraine onset before CHC use (72%) (*p* < 0.02). Comorbidities were rare, except dysmenorrhea. The majority of migraine attacks in CHC users occur during the hormone-free interval. Similar as in the natural cycle**,** hormone-withdrawal migraines in CHC users are very intense and the response to acute medication is less good, especially in those women, who developed migraine before CHC use.

## Background

Combined hormonal contraceptives (CHC) can trigger migraine onset or worsen the course of migraine [1–3]. A typical trigger for headache attacks is hormone withdrawal during the monthly hormone-free interval (HFI), which is also the time frame during which withdrawal bleeding occurs. To date there is little knowledge about characteristics of migraine in CHC users in comparison to nonusers. It is also unknown, if migraine phenotypes differ between CHC users, who had their first migraine attacks prior to the initiation of CHC, or afterwards. The presence of associated gynaecologic comorbidities, like dysmenorrhoea and endometriosis could also modify migraine features [4–9]. Severe dysmenorrhea has a prevalence of around 20% and may be associated with endometriosis and chronic pelvic pain [4–7, 9, 10]. Another well-known comorbidity of migraine and potential adverse event in a subset of CHC users is depression [11–13]. According to the ICHD-3 Appendix Menstrually related migraines (MRM) are attacks on day 1 ± 2 of the menstruation in at least two of three cycles [14]. They are without aura. Migraine attacks occur also at other times of the menstrual cycle, while in women with pure menstrual migraine (PMM) attacks occur only at bleeding days 1 ± 2. Those definitions are not only used for the natural cycle but also for hormone withdrawal bleedings as those in the pill break of CHC users. However, a recent study demonstrated that in CHC users, migraine attacks in relation to bleeding occur most frequently on days − 1 to 4 [15]. While estrogen withdrawal is a major pathomechanism of hormone-withdrawal headaches, if has been shown that continuous use of progestins can exert a positive impact on menstrual and non-menstrual migraines [16].

Hormone withdrawal in CHC users is much more abrupt in comparison with the smoother decline of hormones at the end of the natural cycle. Consequently, also the menstrually related migraines (MRM) in CHC users might differ with regard to onset, intensity, and response to pain medication from MRM in the natural cycle. A better understanding of migraine features in CHC users and awareness for gynaecological comorbidities might have an impact on treatment decisions regarding choice of prevention and the choice of hormones used to treat migraine attacks during the HFI. Progestins used in contraception can exert a positive impact on dysmenorrhea and endometriosis, but also on the course of migraine [17–20].

The present study was carried out to identify migraine features in women who use CHC and to compare these features between individuals with migraine onset before or after first CHC use. Furthermore we aimed to select information on comorbidities like dysmenorrhea, endometriosis and depression, as well as on migraine onset, family history and age at menarche. The latter is frequently mentioned as a typical age associated with migraine onset. With the diary part of the study we aimed to compare migraine frequency, pain intensity, and use of pain medication in the three weeks of pill use and the HFI between both groups.

## Methods

This prospective trial was conducted at the Clinic for Reproductive Endocrinology, University Hospital Zurich, where one of the authors (GSM) runs a clinic for hormonal migraine. Data for this convenience sample were collected from November 2017 to May 2019. We recruited participants in the clinic and through advertisement. Women were contacted by phone to explain the project and to screen for eligibility. Premenopausal women aged 18–50 years were included, if they had a history of MRM, as defined in the ICHD-3 during in at least two of three cycles and used any type of CHC with a monthly hormone-free period of 7 days (21/7 regimen). Women were included if they agreed to participate in an interview only or if they agreed to both, an interview and daily conducted diaries for three cycles. Exclusion criteria comprised CHC use in flexible or extended cycles, use of other hormones and migraine during CHC use but not in the HFI. All women gave written informed consent.

Data were collected during a semi-structured telephone interview performed by two residents who are familiar with the International Classification of Headache Disorders (ICHD-3) under supervision of a senior gynaecological headache specialist (GSM) [14]. We aimed to perform 50 complete interviews. We collected information on age at migraine onset, frequency, severity (numerical rating scale from 1 to 10), duration and localization of headaches, aura triggers, associated symptoms, family history as well as use of acute medication and prophylactic medication. Furthermore we explored comorbidities with a focus on depression, dysmenorrhea, endometriosis, and other chronic pain conditions. Age at menarche, first start of use of a CHC and the type of CHC used at present were documented. All women were asked to conduct a prospective headache diary over three CHC cycles (84 days) to document migraine days, pain intensity and the amount of pain medication used during the HFI and during pill use. Additional diary data were used for a separate project [15]. The hormone-free days and days with uterine bleeding were also recorded. For the diaries, a simple pain score from 0 to 3 was used: 0 = no, 1 = mild, 2 = moderate and 3 = severe pain in accordance with ICHD-3 [14]. Diaries were send out per email and started on the first day of pill use. To facilitate comparison with the natural cycle, which starts with hormone withdrawal and bleeding we present our data starting with the HFI at days 1–7. Weeks 2, 3, and 4 were the weeks with daily CHC use. Women, who had agreed to conduct a diary returned the diary per email after each cycle. All data were analysed for women with migraines onset before start of CHC (group 1) and migraines onset after having started CHC (group 2). The study has been approved by the regional ethics committee (KEK-stV-Nr 2016-01791) and was registered on clinicaltrials.gov: NCT04012593.

### Statistical analyses

The programmes IBM SPSS Statistics 22 and Excel 2016 were used for statistical analyses. Continuous demographic and clinical characteristics were presented as means ± standard deviation (SD). Frequencies and percentages were used to describe dichotomous characteristics. If women answered a question with ‘I do not know’ this answer was not included in the analysis. Mann–Whitney *U* Test or Fisher’s exact test were used to compare demographic data and characteristics between women with migraine already existing before CHC use (group 1) and women, whose migraine onset was after first use of CHC (group 2). In the subgroup of participants, who conducted a daily diary migraine frequency, intensity (pain score 0–4) and number of pain medications are reported weekly during the HFI. For weeks 2–4, the mean values were divided by 3 to receive a weekly number for this period, which allows comparison with the one week HFI. Mean pain score was calculated as sum/week. Also here the value was divided by 3 to get a weekly mean value to allow comparison between the HFI and the phase of hormone use. *p* values ≤ 0.05 were considered as statistically significant.

## Results

**Fig. 1:**
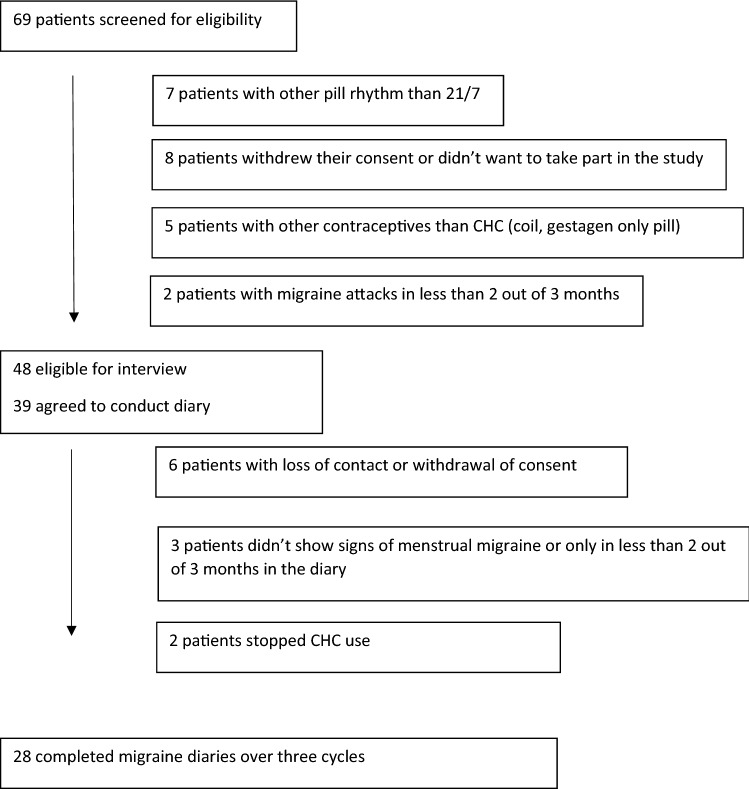
Flowchart: inclusion and exclusion process

Among the 69 patients screened, 48 were eligible for the study and were interviewed. Out of thirty-nine women, who agreed to conduct a diary after the interview, 28 completed study diaries over three cycles (Fig. 1). Only three individuals had only menstrually related attacks i.e. pure menstrual migraine (PMM), while all others experienced in addition attacks during use of the CHC. The three persons with PMM were all in group 2. Their data were not included into the analyses of the first two questions in Table 2 (pain intensity and response to pain medication in menstrually related attacks). Baseline characteristics are presented in Table 1 and more detailed headache features, gynaecologic characteristics and comorbidities in Table 2. Demographic data and baseline migraine features did not differ between women who participated in the interview only and those who also conducted the diary (Table 3).

**Table 1:** Comparison of demographic and clinical characteristics of patients with migraine existing before start with combined hormonal contraception (CHC) (group 1) and women whose migraine started after having initiated CHC (group 2)

Characteristics*	All *n* = 48	Group 1 *n* = 22	Group 2 *n* = 26	*p* value
Age (years) mean (SD)	26.5 (5.0)	28.6 (6.3)	27.3 (4.4)	0.29
Size (cm) mean (SD)	165 (5.3)	164 (6.0)	166 (5.0)	0.2
Weight (kg) mean (SD)	60.3 (14.3)	58.1 (16.0)	62.2 (13.0)	0.049
Age at first menstruation (year) mean (SD)	13.0 (1.2)	12.7 (1.2)	13.2 (1.19	0.13
Age at first migraine attack (year) mean (SD)	17.9 (5.7)	14.2 (4.9)	21.0 (4.4)	0.001
Ages at first start with CHC mean (SD)	17.4 (2.7)	16.7 (1.4)	18.8 (3.5)	0.39
Number of migraine days/months at age < 20 years mean (SD)	1.9 (2.4)	2.3 (2.5)	1.6 (2.3)	0.03
Number of migraine days/months now mean (SD)	4.2 (2.7)	5.0 (3.1)	3.5 (2.1)	0.048
Pain score of attacks on a scale of 1–10 mean (SD)	6.5 (1.6)	6.4 (1.9)	6.7 (1.3)	0.78
Migraine with aura: yes *n* (%)	9 (18)	4 (18)	5 (19)	0.98
Aura only once *n* (%)	31 (64)	14 (63)	17 (65)	0.53
Duration of attacks (h) mean (SD)	27.7 (34.9)	25.2 (30.0)	29.8 (38.8)	0.86
Dysmenorrhoea % (*n*)	75.0 (36)	63.6 (14)	84.6 (22)	0.049
Smoking: yes % (*n*)	3.8 (8)	1.3 (6)	0,5 (2)	0.07
Sport: h/week mean (SD)	3.2 (2.6)	3.6 (3.0)	2.7 (2.1)	0.51

*Questions answered with: I do not know, or I do not remember were excluded from the final analyses

**Table 2:** Migraine features and clinical characteristics of patients with migraine existing before start with combined hormonal contraception (CHC) (group 1) and women whose migraine started after having initiated CHC use (group 2)

Migraine feature *n*	All *n* = 48	Group 1 *n* = 22	Group 2 *n* = 26	*p* value
Menstrually related attacks are more painful than others* % (*n*)	57.5 (26)	72.7 (16)	43.4 (10) 23*n*	0.015
Menstrually related attacks respond good to pain medication* % (*n*)	47.8 (22)	31.8 (7)	65.2 (15)	0.05
Pain location: unilateral mostly or always % (*n*)	95.1 (39)	95 (19)	95,2 (20)	0.99
Family history positive % (*n*)	72.9 (35)	86.4 (19)	61.5 (16)	0.05
Medication overuse headache in the past % (*n*)	10.4 (5)	10.0 (2)	11.5 (3)	0.62
Status migrainosus ever % (*n*)	41.7 (20)	47.6 (10)	38.5 (10)	0.42
Triptan use as acute medication % (*n*)	40.9 (18)	27 (5)	54.2 (13)	0.04
Ever use of prophylactic agents % (*n*)	8.9 (6)	3.2 (1)	19.2 (5)	0.76
Non-hormonal trigger yes % (*n*)	81.1 (39)	81.8 (18)	80.8 (21)	0.61
Associated symptoms % (*n*)	87.5% (42)	86.4 (19)	88.5 (23)	0.58
Nausea % (*n*)	62.5 (30)	54.5 (12)	69.0 (18)	0.22
Vomiting % (*n*)	29.2 (14)	18.2 (4)	38.5 (10)	0.20
Photophobia % (*n*)	64.6 (31)	63.6 (14)	65.4 (17)	0.57
Phonophobia % (*n*)	45.8 (22)	36.4 (8)	53.8 (14)	0.26
Endometriosis % (*n*)	0 (0)	0 (0)	0 (0)	0.99
Depression % (*n*)	4.3% (2)	0 (0)	7.7 (2)	0.31
Other types of chronic pain % (*n*)	4.3% (2)	0 (0)	7.6 (2)	0.41
History of traumatic brain injury, meningitis, concussion % (*n*)	0 (0)	0 (0)	0 (0)	1.0
History of stroke % (*n*)	0 (0)	0 (0)	0 (0)	1.0

*Three participants from group 2 with PMM were excluded from this analysis

**Table 3:** Comparison of demographic data and migraine characteristics in women participating in the interview only and participating in the interview and diary part of the study

Characteristic	Interview only *n* = 20	Responders diary and interview *n* = 28	*p*
Age (years) mean (SD)	27.1 (5.2)	27.9 (5.3)	0.74
Size (cm) mean (SD)	166.6 (4.9)	165.1 (5.9)	0.32
Weight (kg) mean (SD)	61.5 (16.7)	59.3 (12.8)	0.45
Smoking number daily mean (SD)	0.5 (1.3)	0.29 (0.9)	0.54
Sport/h/week mean (SD)	3.6 (3.0)	2.8 (2.2)	0.31
Migraine before CHC start % (*n*)	45 (9)	46.4 (13)	0.87
Age at first menstruation (year) mean (SD)	12.9 (1.3)	13.1 (1.1)	0.46
Age at first migraine attack (year) mean (SD)	17.8 (5.4)	18.0 (6.0)	0.93
Number of migraine days/months at age < 20 years mean (SD)	2.0 (2.4)	1.8 (2.4)	0.62
Number of migraine days/months now mean (SD)	4.1 (3.1)	4.2 (2.4)	0.85
Pain score of attacks on a scale of 1–10 mean (SD)	6.1 (1.9)	6.9 (1.3)	0.23
Are menstrual attacks more painful % (*n*)	52.6 (10)	57.1 (16)	0:79
Migraine with aura: yes % (*n*)	100 (20)	100 (27)	0.71
Aura only once % (*n*)	80 (12)	68 (19)	0.68
Duration of attacks (h) mean (SD)	21.8 (19.4)	29.8 (34.7)	0.31
Dysmenorrhoea % (*n*)	78.9 (15)	75.0 (21)	0.92
Pain unilateral always or mostly % (*n*)	94.7 (18)	95.5 (21)	0.91
Family history for first- or second-degree relative yes % (*n*)	65.0 (13)	67.9 (19)	0.23

Mean age of the participants was 26.5 ± 5 years, mean age at the first migraine attack was 17.9 ± 5.7 years, the mean age at the first menstrual bleeding was 13.0 ± 1.2 years and the mean number of migraine days/month was 4.2 ± 2.7. Nineteen percent of patients reported a history of more than one auras. In comparison to women who developed their migraine after start with CHC (group 2 *n* = 26), women with migraine before CHC start (group1 *n* = 22) were younger at migraine onset, suffered less frequently from dysmenorrhoea and had more monthly migraine days (Table 1). They also reported more often a positive family history (FH) for migraine (Table 2). Furthermore a higher percentage of individuals in group 1 experienced MRM as more painful and less responsive to rescue medication. Interestingly, the percentage of triptan users was nevertheless lower in comparison to group 2. Diary data confirmed the total number of migraine days/month (4.11 ± 2.8) and showed a trend to a higher frequency in group 1 (*p* < 0.07). The weekly pain score differed between groups only during the HFI (*p* < 0.02) (Table 4). In addition there was a trend towards a higher number of migraine days (*p* < 0.06) and pain medications used during this period (*p* < 0.07) for group 1.

**Table 4:** Diary-based migraine frequency, pain score and days with pain medication in patients with migraine existing before start with combined hormonal contraception (CHC) (group 1) and women whose migraine started after having initiated CHC (group 2)

	Migraine days			p**	Days with pain medication			p**	Mean Pain score			p**
	All	Group 1	Group 2			Group 1	Group 2			Group 1	Group 2	
Complete cycle (28 days)	4.1 ± 2.8	4.5 ± 2.2	3.8 ± 3.2	0.07	3.2 ± 3.0	3.5 ± 2.6	3.0 ± 3.3	0.15	8.3 ± 7.7	9.0 ± 4.5	7.7 ± 9.5	0.03
Week 1 (mean SD) *Hormone-free interval*	2.1 ± 1.6*	2.4 ± 1.4*	1.9 ± 1.7*	0.06	1.6 ± 1.3*	1.7 ± 1.3*	1.5 ± 1.3*	0.35	4.4 ± 3.3*	5.0 ± 2.9*	3.8 ± 3.6*	0.02
Weekly mean of weeks 2–4 (mean SD) *Pill use*	0.7 ± 1.0	0.7 ± 0.5	0.6 ± 0.9	0.34	0.6 ± 0.8	0.6 ± 0.6	0.5 ± 1.0	0.53	1.3 ± 2.2	1.3 ± 1.2	1.3 ± 2.8	0.43

*p < 0.001 week 1 compared to mean of weeks 2–4

**p between groups

Among the most frequent non-hormonal triggers were stress (58.3%), sleep deficiency (35.4%) and weather changes (18.8%). Less than 10% of the individuals mentioned odours, long working time spend at a computer screen or hunger. The subgroups did not differ with regard to migraine triggers or associated symptoms. Associated symptoms reported from more than 10% of the participants comprised nausea, vomiting photophobia and phonophobia (Table 2). Only six women had ever used a prophylactic substance, mostly (*n* = 5) in form of riboflavin or magnesium or both. Only one person had a history of a pharmacological prophylaxis, topiramate.

None of the interviewed participants had a history of meningitis, traumatic brain injury, or concussion.

Apart from dysmenorrhoea, migraine associated medical conditions were rare in both groups of young women (no endometriosis, two women with a history of depression).

Migraine onset occurred within ± 2 years in relation to menarche in 63% of the women in group 1. Within CHC users who had not experienced migraine earlier 38.5% experienced the onset of migraine within 2 years and 57.7% within 4 years after start of this contraceptive method.

## Discussion

Interview data demonstrated, that most migraine days in CHC users occurred during the HFI. Around 50% of CHC users report, that these menstrually related migraines are more painful than other migraine attacks and respond less good to pain medication. Interestingly, migraine features and family history differed between individuals who already suffered from migraine before starting a CHC (group 1) and those who developed it after the initiation of CHC (group 2). Women in group 1 reported a higher frequency of monthly migraine days more painful menstrually related attacks, inferior response to pain medication, a higher pain score and use of more pain medication in the hormone-free week. Also, a positive family history for migraine was more common in group 1. In the diaries the pain score, the number of migraine days and the number of days with pain medication were higher in the HFI for the total group and within the two subgroups in comparison with the weeks of pill intake.

In accordance with our data, trials comparing menstrual and non-menstrual migraine report consistently, that MRM are more severe, of longer duration and more resistant to treatment compared to non-menstrual attacks [21–25]. Up to date, it was not clear if hormone withdrawal migraines in CHC users have the same characteristics. Based on diaries conducted over 2 months, Coffee et al. did not find any difference in daily headache scores in 21 CHC users, but did not distinguish non-MRM and MRM [26]. In another trial, 55% of 20 CHC users with pure menstrual migraine, rated their attacks as severe [27]. It is unclear, if women in this trial had already suffered from migraine before the initiation of hormonal contraception. In a large retrospective cohort study, a similar percentage (52.3%) of hormonal contraceptive users reported a worsening of migraine in temporal relation to the menstrual bleeding [28]. This is in line with our finding of 57% women describing MRM as more painful. Notably this percentage was significantly higher in women with migraine before CHC start (73%). The overall pain score reported in the interviews for all migraines did not differ between groups.

In our study, the mean age at the first migraine attack was 17.9 years and significantly lower (14.2 years) in women who developed migraine before CHC start. In other trials on hormonal migraines, this age ranges from 16 to 25 years. No information is given on the age at menarche [29–32]. The mean age of our participants was low (26.5 years) in comparison to other studies on MRM, which might reduce the risk of recall bias concerning the age at menarche and the age at the first migraine attack [24, 27, 32, 33]. In group 2, the mean time frame between CHC start and migraine onset was 2.2 years. Insofar it is open in how many women a causal relationship between CHC use and migraine onset exists.

Onset of migraine had occurred within 2 years before or after menarche in 63% of the individuals of group 1. We present data in this 2-year interval, as the hypothalamic–pituitary system starts to release first irregular impulses and later more regular impulses already around two years prior to the first bleeding [34]. Our data indicate that many, but by far not all, hormone-related migraines start in this period with the most relevant hormonal alterations during puberty. In a survey including 80 women (mean age 33.6 years) with episodic migraine around 50% indicated that migraine onset had occurred within 1 year after menarche [33]. In our study, only two of 15 women reported migraine onset in this time span. Difficulties to recall age at the first menstruation up to 20 years later might at least partly explain the differences to our results.

Despite the difference in mean age compared to other trials (35.3 years; 38.3 years), the frequency of migraine days/month in our trial with CHC users (4.2) was similar to that in other reports (4.4 and 5.0) investigating MRM [30, 32]. Migraine frequency was, however, significantly higher in group 1 (Tables 1 and 4). In both groups, the monthly frequency was twofold compared with the frequency reported below the age of 20 years. From our study design, it cannot be inferred, if there this is a physiologic age-related increase in migraine days or to some extent a causal relationship with CHC use. A rapid age-related doubling of migraine frequency does not seem to be very probable given the data on migraine frequency and age in the two aforementioned studies. However, having 1–2 more migraine days per month can be related to increased disability, if those migraine days cannot be treated successfully. Especially if these are attacks in the HFI. The diary data confirmed, that the pain score in this week is especially high (*p* < 0.001) and even more high in group one (*p* < 0.02). In respect of migraine aura, we did not see differences between both groups (Table 1). This suggests, that the predisposition to migraine aura is not influenced by the intake of CHC. However, it is striking that patients with possible migraine aura are still prescribed CHC despite the potential risk of thromboembolic events [35, 36]. We would suggest that gynecologists should actively ask for migraine aura prior to discussing contraception with their patients. Estrogen-containing contraceptives should be avoided in patients with migraine aura.

Eighty-six percent of the individuals in group 1 had a positive family history for migraine, which is in line with findings from epidemiologic studies demonstrating an association of a positive family history with an early onset of migraine [37, 38]. Furthermore, depression was found in 57% of the migraineurs with FH. In our trial only two women reported to have experienced a depressive episode in the past. None of them had used antidepressants. Psychiatric disorders and musculoskeletal disorders are significantly more common in patients with chronic than in those with episodic migraines. Older age and longer duration of disease have an impact on the prevalence of the comorbidities, what explains the low rate of this conditions in our trial [39]. A history of dysmenorrhoea was reported by 76.6% of women in our study. This percentage was even higher in group 2, in which on the other hand fewer women had a positive family history. Spierings et al. found in women with menstruation-sensitive migraine that 43.8% suffer from dysmenorrhoea and 10% from endometriosis [33]. Dysmenorrhoea is more frequent in younger women. In an Australian survey, 93% of teenagers aged 16–18 years experienced some degree of pain during menstruation. Insofar the numbers in our study regarding this comorbidity are in the range of young women without migraine. In the same Australian survey, evaluating comorbidities of dysmenorrhea, stabbing pelvic pain was associated with migraine (OR of 2.3). About one-fifth (21.1%) of the responders with dysmenorrhoea reported headaches on 15 or more days per month [4]. The severity of dysmenorrhea was not quantified in our study and use of CHC users is typically associated with reduced pain severity of dysmenorrhea. Endometriosis had not been diagnosed in any of our participants. Nevertheless, a subset of the women in our study suffering from dysmenorrhea might have this condition.

In both groups, most migraine attacks were described as unilateral. Triptan use was more frequent in group 2, also migraine intensity did not differ between groups. Furthermore, in group 1, only one person had ever tried to use prophylactic substances, while this was the case in five persons of group 2. It can only be speculated that migraineurs of group 1 might have developed better coping strategies during their longer history of migraine or by having taken family members as role models [40].

Migraine triggers were reported by about 80% of the participants, which is similar to the 76% of migraineurs reporting triggers in a study with 1750 women suffering from episodic headache [41]. In line with other studies, stress and sleep deficiency were by far the mostly reported trigger [42–44]. No difference was found between the groups.

Associated symptoms were frequent (87%) and did not differ between groups. The frequency is in congruence with findings from other authors for MRM in not CHC users [29, 30, 32].

Altogether we found in our sample that most migraine days in CHC users occur during the HFI. Migraines are mostly unilateral and triggers do not differ from those reported from other authors for episodic migraine. The menstrually related attacks are more painful and less responsive to acute pain medication. Therefore gynaecologists and neurologists should discuss other contraceptive options, like modern progestin-only pills, which would also have a positive impact on dysmenorrhoea and a potentially not yet diagnosed endometriosis. The group of young women with migraine before CHC use reporting earlier migraine onset, higher migraine frequency, higher pain scores and a stronger family predisposition might belong to a distinct clinical entity. These persons should even more be advised to use a contraceptive with rather a beneficial effect on their migraines, like the progestin desogestrel [19, 45, 46]. Our data did not allow to clarify, if the considerable increase in migraine days after age twenty might indeed be related to the use of CHC or if this rather corresponds to the natural course of migraines in these young women.

### Strength and limitations

A limitation of this project is the sample size, which is smaller as compared to web-based surveys or surveys using self-completed questionnaires without any instruction for the individuals enrolled. Our sample size is within the range of that of other interview and diary-based studies addressing migraine features [21, 27, 29, 30, 33, 47, 48]. A strength is, that the interview data were collected from experienced headache specialists, insofar a higher correctness of responses can be expected in comparison with self-completed questionnaires or online surveys. In addition a bias by misunderstanding questions is unlikely. Furthermore, the high percentage of women who recorded headache characteristics in prospective daily diaries contributes to the quality of the data. The number of migraine days did not differ between women participating solely in the interview and those keeping a prospective diary over three cycles. The diaries over 3 months confirmed the diagnosis of MRM in at least two of three cycles. The strict inclusion/exclusion criteria, checked by a skilled doctor reduced the number of eligible women but contributed to a higher validity. For group 2, it remains open, if the use of CHC is the reason why these women developed a migraine. Strengths of the study include the exact definition of the type of hormonal contraception used, the exclusion of progestin-only methods or hormone-releasing-intrauterine devices and the inclusion of women only with withdrawal headache in at least two of three cycles as required in the ICHD. In addition we ensured, that results are not biased by women suffering from posttraumatic or post-inflammatory headaches.

## Conclusion

Migraines in CHC users occur mostly in the hormone-free interval. They are typically unilateral and associated symptoms and triggers do not differ from those described by other authors. The MRM during the HFI are also in CHC users more intense and less responsive to acute medication. This is more pronounced in women with early migraine onset, a positive family history and migraine before use of CHC. The frequency of typical comorbidities was low except for dysmenorrheoa.

## Data Availability

The datasets used for this study are available from the corresponding author on reasonable request.

## Abbreviations

CHC: Combined hormonal contraceptives
HFI: Hormone-free interval
ICHD: International Classification of Headache Disorders
MRM: Menstrually related migraine
PMM: Pure menstrual migraine
